# P-1049. Retrospective Analysis on the Efficacy and Adverse Effects of Antifungal Prophylaxis in Patients with Peritoneal Dialysis-Associated Peritonitis

**DOI:** 10.1093/ofid/ofae631.1239

**Published:** 2025-01-29

**Authors:** Vidal Luchana, Shivanthidevi Gandhi, Thien-Ly Doan, Barbara Kamel, Sumeet Jain, Rubab Sohail, Cristina Sison, Henry Donaghy

**Affiliations:** Long Island Jewish Medical Center, New Hyde Park, New York; North Shore University Hospital, New Hyde Park, New York; Long Island Jewish Medical Center, New Hyde Park, New York; Northwell Health, New Hyde Park, New York; North Shore University Hospital, New Hyde Park, New York; Northwell Health, New Hyde Park, New York; Feinstein Institutes for Medical Research, Manhasset, New York; Northwell Health, New Hyde Park, New York

## Abstract

**Background:**

Fungal peritonitis causes significant mortality for patients on peritoneal dialysis (PD). Antibiotic exposure is a major risk factor for fungal peritonitis amongst PD patients. The 2016 and 2022 International Society for Peritoneal Dialysis (ISPD) Guideline recommends the use of antifungal prophylaxis in PD patients on antibiotics. There is still no consensus on the optimal approach to prevent secondary fungal peritonitis. This study's objective was to evaluate the impact of antifungal prophylaxis on patients with PD-associated peritonitis as secondary prevention for fungal peritonitis.

**Figure:**
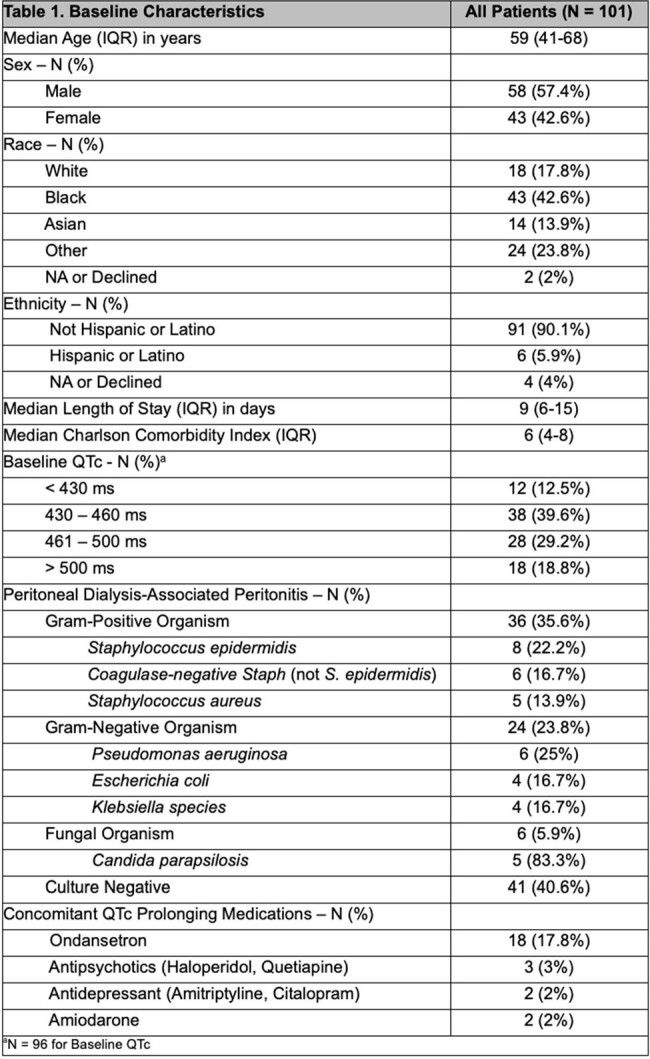

**Methods:**

This is a retrospective cohort study of patients on concomitant antifungal prophylaxis and those with no antifungal prophylaxis who had confirmed or suspected PD-associated peritonitis while on antibiotic therapy from 2018 to 2023. Descriptive analysis was used to compare the outcome of development of secondary fungal peritonitis. Side effects such as QTc prolongation are also described.

**Figure:**
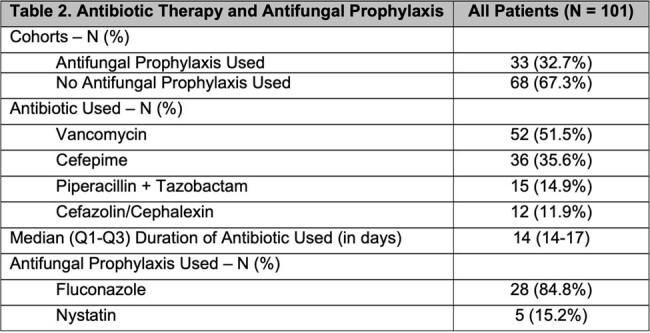

**Results:**

Of the 101 patients with PD-associated peritonitis, there were 36 (35.6%) gram-positive infections, 24 (23.8%) gram-negative infections, 6 (5.9%) fungal infections, and 41 (40.6%) culture negative peritonitis. The most common isolates were *Coagulase-negative Staphylococcus* (14), eight of which were *Staphylococcus epidermidis*, followed by *Pseudomonas aeruginosa* (6). Among the 101 patients, 33 (32.7%) were co-prescribed with antifungal prophylaxis while 68 (67.3%) did not. Fluconazole 100 mg daily was the most common antifungal agent used (84.8%). Over a 1-year period, two patients developed secondary fungal peritonitis in patients with concomitant antifungal prophylaxis (6.1%; 95% CI: 0.74% to 20.23%), compared to 1 patient (1.5%; 95% CI: 0.037% to 7.92%) in patients without antifungal prophylaxis. Among 28 patients on fluconazole, 10 had repeat ECGs. Of these, 7 patients had a QTc greater than 460 ms (70%; 95% CI: 34.8% to 93.3%), and 3 were greater than 500 ms (30%; 95% CI: 6.7% to 65.2%).

**Figure:**
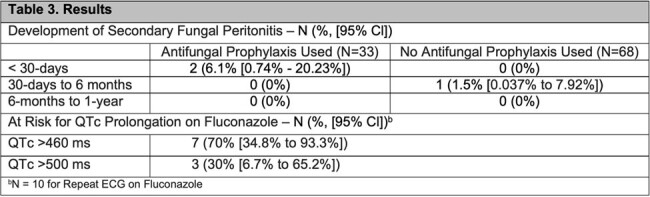

**Conclusion:**

Antifungal prophylaxis in the setting of PD-associated peritonitis does not correlate to fewer episodes of secondary fungal peritonitis. Increased QTc with azole use is a potential risk factor for cardiac arrhythmia.

**Disclosures:**

**All Authors**: No reported disclosures

